# High Density Lipoprotein and Its Precursor Protein Apolipoprotein A1 as Potential Therapeutics to Prevent Anthracycline Associated Cardiotoxicity

**DOI:** 10.3389/fcvm.2020.00065

**Published:** 2020-04-28

**Authors:** George E. G. Kluck, Kristina K. Durham, Jeong-Ah Yoo, Bernardo L. Trigatti

**Affiliations:** ^1^Department of Biochemistry and Biomedical Sciences, Thrombosis and Atherosclerosis Research Institute, McMaster University and Hamilton Health Sciences, Hamilton, ON, Canada; ^2^Faculty of Health Sciences, Institute of Applied Health Sciences, School of Rehabilitation Sciences, McMaster University, Hamilton, ON, Canada

**Keywords:** HDL, ApoA1, anthracyclin, chemotherapy, cardiotoxicity, cardioprotective, doxorubicin

## Abstract

Cardiovascular disease and cancer are the leading causes of death in developed societies. Despite their effectiveness, many cancer therapies exhibit deleterious cardiovascular side effects such as cardiotoxicity and heart failure. The cardiotoxic effects of anthracyclines such as doxorubicin are the most well-characterized of cardiotoxic anti-cancer therapies. While other anti-neoplastic drugs also induce cardiotoxicity, often leading to heart failure, they are beyond the scope of this review. This review first summarizes the mechanisms of doxorubicin-induced cardiotoxicity. It then reviews emerging preclinical evidence that high density lipoprotein and its precursor protein apolipoprotein A1, which are known for their protective effects against ischemic cardiovascular disease, may also protect against doxorubicin-induced cardiotoxicity both directly and indirectly, when used therapeutically.

## Introduction

**Figure 1:**
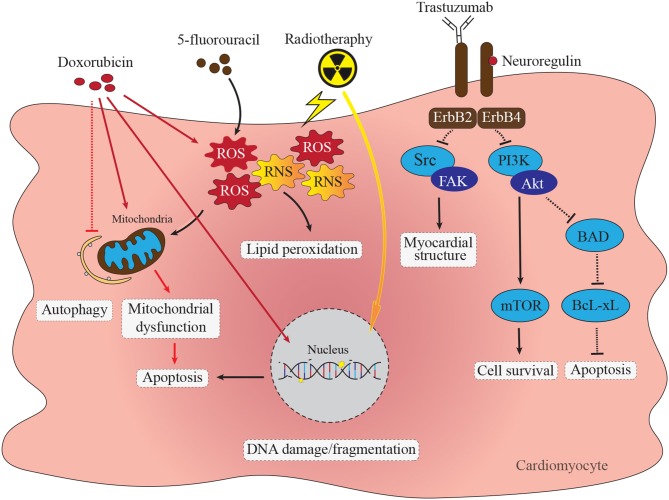
Direct cardiotoxic effects of anti-cancer therapies in cardiomyocytes. Doxorubicin cardiomyocyte cytotoxicity through diverse pathways including DNA damage, accumulation of ROS and RNS, mitochondrial dysfunction, apoptosis and impaired autophagy. Inhibition of ErbB2/4 receptor complex with trastuzumab impacts several signaling pathways resulting in impaired protein synthesis and myocardial structure via the Src/Fak pathway (6). Trastuzumab mediated supression of ErbB2/4 stimulated PI3K-Akt signaling impacts cell survival via mTOR pathway as well as increases apoptosis via BAD/BcL-xL pathway (7). Chemotherapy with fluoropyrimidines, such as 5-fluorouracil (5-FU) cause increase in formation of ROS and RNS leading to mitochondrial dysfunction and activation of caspase-3 and apoptosis (8). Radiotherapy leads to ROS and RNS formation, mitochondrial dysfunction and single-stranded DNA breaks leading to apoptosis activation (9).

Advances in cancer treatment over the past few decades have led to substantial increases in cancer survivorship (1, 2). As cancer-related survival has improved, an unexpected increase in premature cardiovascular events, including myocardial ischemia, myocardial infarction, congestive heart failure (HF), QT interval prolongation, hypertension, and stroke has occurred (3, 4). A major contributing factor to cardiovascular outcomes is cardiotoxicity related to antitumor drugs, which may become apparent acutely during treatment, or often, well after treatment has ended (4, 5). In general terms, cancer therapy related cardiotoxicity ultimately leads to pathological alterations in the cardiac muscle tissue (5). Many different classes of chemotherapeutic agents have cardiotoxic effects. Anthracyclines such as doxorubicin (DOX) are, perhaps, the most well-studied cardiotoxic chemotherapeutic agents. They have a number of direct cardiotoxic effects, including DNA damage, interfering with mitochondrial function, induction of reactive oxygen species (ROS), alterations in autophagy and induction in apoptosis. Mechanisms of anthracycline induced cardiotoxicity will be discussed in more detail below. Other cardiotoxic chemotherapeutic agents/treatments include fluoropyrimidines, such as 5-fluorouracil, biologicals, such as trastuzumab and radiation therapy, all of which impact cardiomyocyte survival by triggering apoptosis through differing, but overlapping pathways (summarized in Figure 1). For example, fluoropyrimidines, such as 5-fluorouracil (5-FU) and capecitabine, are used to treat different types of tumors, especially those that appear in the head, neck and breast (10, 11). In the case of these agents, cardiotoxicity appears to be due to both direct toxic effects of these drugs on cardiomyocytes, through the generation of reactive oxygen species (ROS) and reactive nitrogen species (RNS), leading to both oxidative and nitrosative stress, and activation of apoptosis and autophagy (12). Furthermore, these agents also appear to have indirect cardiotoxic effects through interaction with the coagulation system and autoimmune responses (8). Trastuzumab, an antibody targeting the Human epidermal growth factor 2 receptor (HER/ErbB2), and used as first choice therapy against breast cancer (13, 14), also inhibits this receptor in cardiomyocytes, affecting myocardial structure and survival pathways, triggering cardiomyocyte apoptosis and leading to asymptomatic decreased left ventricular ejection fraction, and eventually heart failure, particularly when it is used in combination with other agents, such as anthracyclines (15, 16). Radiation therapy also triggers cardiotoxicity through induction of DNA damage as well as ROS in cardiomyocytes, again affecting cell survival pathways and triggering apoptosis (17). Radiation therapy induced cardiovascular effects may manifest as pericarditis, coronary artery disease, myocardial infarction, valve heart disease, changes in rhythm, silent myocardial ischemia and damage to the conduction system (18). The risk of heart disease is mainly related to the total radiation dose and the volume of the heart receiving radiation (19).

As a result of these cardiotoxic effects, the mode of administration, time and dose of these treatments and the presence of pre-existing comorbidities such as cardiovascular and liver disease, diabetes mellitus and hypertension are important factors to be taken into consideration upon treatment (20). Furthermore, long term follow-up for cardiovascular complications is important, particularly in those patients with pre-existing cardiovascular risk factors (21–23). Understanding the mechanisms by which chemotherapeutic agents cause cardiotoxicity, as well as the identification of pathways that can be targeted in the cardiomyocyte to selectively mitigate chemotherapy induced cardiotoxicity is of fundamental importance in decreasing the undesirable impact on normal tissues and improving cancer treatment outcomes.

High density lipoproteins (HDL) have long been associated with cardioprotection, with a major focus being on arteriosclerosis-associated ischemic heart disease and stroke (24–28). This has largely been associated with its role in the transport of cholesterol from the artery wall to the liver for excretion or recycling, a process called reverse cholesterol transport (RCT) (29). However, recent advances in our understanding of HDL properties and biological functions have revealed HDL associated functions extending beyond cholesterol transport, including direct cytoprotective effects on a number of cell types including cardiomyocytes (30, 31). These advances provide key insights into the potential of exploiting these cytoprotective properties for therapeutic approaches to mitigate chemotherapy associated cardiotoxicity.

Here-in, we will first review the mechanisms by which anthracyclines, the most well-characterized of the cardiotoxic chemotherapeutic agents, induce cardiotoxicity, focusing on DOX as a well-studied anthracycline. While other anti-neoplastic drugs also induce cardiotoxicity, often leading to heart failure, they are beyond the scope of this review. We will then review recent advances in our understanding of HDL functions and properties that can be exploited to mitigate DOX-associated cardiotoxicity. These include understanding the mechanisms by which HDL induces cytoprotective responses in cells including cardiomyocytes, the ability of HDL and synthetic particles based on it to encapsulate DOX and serve as delivery vehicles, and findings that therapeutic treatment with HDL's major structural protein, apolipoprotein A1 (ApoA1) appears to have direct antineoplastic effects in preclinical tumor models (32–34).

## Mechanisms of Anthracycline Induced Cardiotoxicity

Anthracyclines are a class of chemotherapeutics commonly prescribed to both adult and pediatric populations (35) and can be used alone or in combination with other cancer treatments. Since its discovery in the late 1960's, DOX (also called adriamycin, the prototypical anthracycline) has become widely prescribed due to its efficacy in treating cancers of both hematologic and solid origin (36, 37). Despite their widespread use, anthracyclines such as DOX are not specific in their cell target and exhibit cytotoxic effects in cardiomyocytes thereby limiting their long-term use due to dose-dependent cardiotoxicity (38). Immediate cardiac side effects of DOX infusion are detectable in the form of arrhythmias (39), and cardiotoxic outcomes can be measured following termination of treatment in both early (weeks to months) and late phases (years). These outcomes range from asymptomatic left ventricular dysfunction, to problematic arrhythmias and severe symptomatic congestive heart failure (37, 38). In a retrospective analysis of three trials of DOX therapy for breast or small cell lung cancers, the estimated cumulative percentage of patients with congestive heart failure was 5% in patients receiving a cumulative DOX dose of 400 mg/m^2^, which increased to 26% at a dose of 550 mg/m^2^, and 48% at 700 mg/m^2^ (40). The incidence of cardiotoxicity is highest within the first year following the termination of chemotherapy in adults, although in cases of childhood cancer, onset of cardiotoxicity has been observed to be delayed in survivors by 4–20 years (40–43).

**Figure 2:**
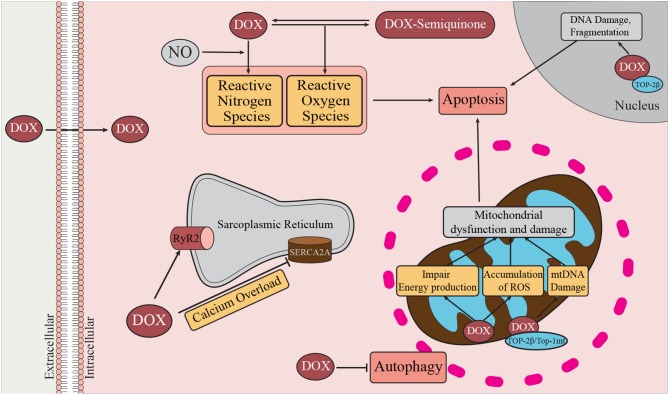
Mechanisms of DOX-induced cardiotoxicity. DOX enters the cell by passive diffusion, and within the cytoplasm undergoes redox cycling resulting in the generation of ROS and RNS (47). Oxidative and nitrosative stress are known to contribute to activation of cell death pathways, such as autophagy, necrosis, and apoptosis. DOX can promote Ca^2+^ overload by inhibiting SERCA2a and transiently enhancing the activity of RyR2. DOX can bind cardiolipin, a major inner mitochondrial membrane lipid. At the mitochondria DOX promotes damage to mitochondrial DNA (mtDNA) through interactions with Top-2β, and Top-1mt. In the nucleus, DOX interacts with Top-2β and DNA to form the Top-2β–DOX—DNA cleavage complex (48). By binding to Top-2β, DOX disrupts the rejoining of DNA leading to the accumulation of double stranded DNA breaks thereby triggering apoptosis (49). Damage to mitochondrial DNA affects mitochondrial biogenesis and contributes to mitochondrial dysfunction and therefore reduced ATP production (50).

A number of pathways have been implicated in DOX-mediated cardiomyocyte cytotoxicity. These include the direct and indirect induction of oxidative stress, DNA damage, and mitochondrial dysfunction. These pathways, along with alterations in homeostatic processes such as autophagy directly and indirectly lead to induction of cell death pathways, including apoptosis and necrosis (44–46) (Figure 2). These pathways have been the subject of recent comprehensive reviews (51–55) and will be summarized in the following sections.

### Oxidative Stress

Oxidative stress is the accumulation of oxygen and nitrogen free radicals resulting when their production exceeds the capacity of anti-oxidant enzymes to detoxify them (56). Oxidative stress has been proposed to be a major contributor to cardiomyocyte death and dysfunction following DOX treatment (56) (Figure 2). DOX can directly induce oxidative stress. The quinone moiety of DOX can act as an electron acceptor which can be reduced by a variety of enzymes to a semi-quinone, with the generation of oxygen free radicals. These can react with proteins, lipids and DNA, resulting in protein dysfunction, lipid peroxidation and DNA damage (50). DOX can also trigger reductions in the activities and expression of antioxidant enzymes, including matrix manganese superoxide dismutase (MnSOD) and glutathione (GSH) peroxidase (57), thereby reducing the antioxidant capacity of the cardiomyocyte. DOX can also interact with nitric oxide (NO) to generate reactive nitrogen species, contributing to nitrosative stress (56). DOX can also contribute to free radical formation and oxidative stress in cardiomyocytes through complex formation with iron (54). DOX treatment also increases accumulation of mitochondrial iron, once again manifesting in increased reactive oxygen and nitrogen species production (50). Dexrazoxane (DRZ) is an iron chelator which has seen limited clinical use to mitigate DOX-associated cardiotoxicity. On the other hand, pre-clinical studies of anti-oxidants have shown limited effectiveness at reducing DOX-associated cardiotoxicity, leading to doubt regarding the role of DOX-induced ROS formation in DOX associated cardiotoxicity (53).

### DNA Damage

DOX also appears to act within the nucleus to trigger cytotoxic effects in cardiomyocytes (Figure 2). DOX can reportedly bind to the cytoplasmic proteasome, which assists in the translocation of DOX to the nucleus by an ATP-dependent nuclear pore-mediated mechanism (58). Topoisomerase (Top)-2β was identified as a molecular mediator of DOX cardiotoxicity (48). Top-2β is located in both the nucleus and mitochondria, and is an important regulator of DNA topology by catalyzing the breaking and rejoining of DNA in order to allow for strands to pass by one another (59). DOX binding to Top-2β and DNA forms the Top-2β–DOX—DNA cleavage complex (48). By binding to Top-2β, DOX reportedly disrupts the rejoining of DNA leading to the accumulation of double stranded DNA breaks thereby triggering apoptosis (49). The importance of this pathway is highlighted by the finding that cardiomyocyte specific deletion of the gene encoding Top-2β protected cultured cardiomyocytes from DOX-induced cytotoxicity and protected mice from DOX-induced cardiotoxicity and heart failure (48). DOX has also been implicated in affecting mitochondrial DNA integrity via similar mechanism of ternary DOX—DNA—topoisomerase complex formation involving Top-2β residing in mitochondria as well as involving a mitochondrial specific topoisomerase, Top-1mt (60, 61).

### Mitochondrial Dysfunction and Damage

Cardiomyocytes are highly dependent on mitochondrial function for energy production and proper contractile function (62–64). As outlined above, DOX can lead to the accumulation of iron within mitochondria and to the iron dependent and independent accumulation of reactive oxygen and nitrogen species which, themselves, can impair mitochondrial energy production (50, 65, 66). Furthermore, as mentioned above, through ternary complex formation between DOX, DNA and mitochondrial topoisomerases (Top-2β and Top-1mt), DOX can damage mitochondrial DNA by inducing double strand DNA breaks (48, 59). This can impair mitochondrial biogenesis as well as further impact mitochondrial function leading to insufficient energy production for the needs of the cardiomyocyte (60, 61).

### Autophagy

Autophagy is a homeostatic mechanism whereby damaged or dysfunctional organelles are recycled to generate substrates for energy production or anabolic processes. Autophagy contributes to normal physiology of the heart and under conditions of acute cardiac stress can promote cardiac survival by releasing energy substrates and breaking down damaged organelles (67). Stimulation of autophagy results in the recruitment of autophagy-related proteins (ATGs) to a specific subcellular site and nucleation of an isolation membrane forming a structure called a phagophore and then an autophagosome, surrounding the damaged target organelle (68). Under conditions of cellular stress, such as nutrient deprivation, the serine/threonine kinase master cell growth regulator, mTOR, is inhibited, resulting in autophagy (69, 70). ATGs that were phosphorylated by mTOR under normal conditions now become dephosphorylated and recruited for autophagosome formation (71). In addition, under conditions of prolonged stress increased autophagic activity can lead to atrophy (decreased size) of cardiac muscle and activation of cell death pathways (67). Tumor-bearing mice display signs of dysregulated cardiac autophagy (increased expression of autophagic markers) and atrophy (32–34), and several research groups have highlighted the importance of autophagy in cardiotoxicity resulting from treatment of mice with DOX (46, 51, 72).

Autophagy is orchestrated by a complex set of regulatory proteins which identify the target for autophagic disposal, form the limiting membrane and orchestrate fusion with lysosomes [reviewed in Li et al. and Koleini and Kardami (51, 52)]. Recent research has demonstrated DOX dysregulates autophagy in cardiomyocytes and that this appears to play an important role in DOX mediated cardiotoxicity. This has been the subject of a number of recent comprehensive reviews (51, 52). DOX appears to exert a dose-dependent disruption of the normal regulation of autophagy: low, clinically relevant doses, replicating chemotherapy, appear to suppress normal levels of basal autophagy, whereas high doses appear to induce autophagy above normal basal levels (51, 52). The impaired autophagy in cardiomyocytes, resulting from chronic low-dose treatment of mice with DOX, has been reported to involve DOX-mediated interference with lysosome mediated degradation of autophagosome contents (73). On the other hand, DOX, particularly at higher doses, appears to lead to an accumulation of PTEN-induced kinase (PINK) 1 and parkin (an E3 ubiquitin ligase) within mitochondria (74). PINK1 and parkin play a key role in regulating the balance between mitophagy and mitochondrial biogenesis; DOX appears to trigger a depolarization of the mitochondrial membrane potential resulting in an accumulation of PINK1 and parkin on mitochondria. PINK1 is a serine/threonine kinase which phosphorylates ubiquitin and allows parkin to ubiquitinate a number of mitochondrial outer membrane proteins, targeting the mitochondria for mitophagy (52, 74). This, possibly together with DOX-mediated interference with lysosome mediated degradation, results in an accumulation of autophagosomes containing mitochondria (52, 73, 74). An emerging consensus appears to be that DOX interferes with autophagic flux, as a result of simultaneous induction of early stages of mitophagy (triggering mitochondrial dysfunction/membrane depolarization, the marking of mitochondria for mitophagy and formation of autophagosomes surrounding dysfunctional mitochondria) and interference with later stages of autophagy (51, 52). As mentioned above, this may be through DOX-mediated interference with lysosome acidification, thereby preventing the lysosomal degradation of contents of autophagosomes (73). Alternatively, others have reported that DOX mediated mitochondrial damage leads to maladaptive activation of phosphatidylinositol 3-kinase (PI3K) γ signaling, which blocked autophagy, and that this was alleviated by cardiac specific PI3K γ inhibition in mice (75). The consequence of these effects appears to be a build-up of damaged/dysfunctional mitochondria and reactive oxygen species, exacerbating DOX-induced oxidative stress, and leading to activation of apoptotic pathways, triggering cell death (51).

### Apoptosis

Apoptosis is a form of programmed cell death involving the induction of a caspase proteolytic cascade ultimately leading to nuclear condensation and fragmentation, phosphatidylserine exposure on the cell surface, formation of apoptotic bodies and their clearance by phagocytes by virtue of recognition of exposed phosphatidylserine. Apoptosis is characterized by the activation of caspase proteolytic cascades leading to the activation of effector caspases, such as caspases 3, 6, and 7 (76, 77). These activate nucleases and proteases which degrade DNA, nuclear, cytoplasmic, and cytoskeletal proteins (76, 77). These effector caspases are activated by two main pathways of apoptosis regulation, the extrinsic and intrinsic pathways. In the extrinsic pathway, ligation of “death receptors” such as the tumor necrosis factor (TNF) α receptor by their extrinsic ligands on the cell surface results in receptor dimerization, bringing together “death domains” leading to the formation of a death inducing signaling complex (DISC) that ultimately triggers the activation of caspase 8, which, in turn, goes on to activate the effector caspases 3, 6, and 7 (77).

On the other hand, mitochondria play central roles in the intrinsic apoptosis pathway, which is regulated by a series of soluble proteins that regulate pore formation in the mitochondrial membrane (76, 77). These factors belong to the Bcl-2 family of proteins and comprise 3 groups: the anti-apoptotic Bcl-2 family members, such as Bcl-2 itself, the pro-apoptotic effectors such as Bax and Bak, and the pro-apoptotic regulators such as Bim. Together these comprise the intrinsic apoptosis pathway. In this pathway, the anti-apoptotic family members bind to and sequester the pro-apoptotic effectors, preventing them from assembling as complexes on the mitochondrial membrane. The pro-apoptotic regulators, in turn, bind to the anti-apoptotic regulators, preventing them from sequestering the pro-apoptotic effectors and, additionally, assist the pro-apoptotic effectors in assembly on the mitochondria, where the pro-apoptotic effectors can form pores in the mitochondrial membrane. This leads to the leakage of mitochondrial cytochrome C and formation of a protein complex, called the apoptosome which serves as a platform for the activation of caspase 9, which can then activate effector caspases 3, 6, and 7 (76). In this intrinsic pathway, the relative amounts of pro-apoptotic effectors, regulators and anti-apoptotic factors determine whether apoptosis proceeds (76). Nuclear DNA damage and induction of oxidative stress and damage to mitochondria are all potent activators of the intrinsic apoptosis pathway (76, 77). In addition, DOX treatment of cardiomyocytes has been reported to result in increased transcription of the pro-apoptotic regulator Bim, thus enhancing the intrinsic apoptotic pathway (78). Additionally, recent work has identified increased expression of death receptors (TNF receptor 1, Fas, death receptor 4, death receptor 5) in human induced pluripotent stem cell derived cardiomyocytes following DOX treatment (44).

### Atrophy

In addition to causing the death of cardiomyocytes, DOX also triggers cardiomyocyte atrophy, or a reduction in cardiomyocyte size. Ultrastructural changes in the myocardium, including myofibril structural disarray and atrophy due to DOX-cardiotoxicity appear well before clinical manifestations (79). The ubiquitin proteasome system regulates cardiomyocyte size by tagging proteins with polyubiquitin chains for subsequent degradation by the proteasome (80). The tagging of target proteins for proteolysis by the ubiquitin-proteasome system involves the assembly of polyubiquitin chains on target proteins mediated by E1 ubiquitin activating enzymes, E2 ubiquitin conjugating enzymes and E3 ubiquitin ligases. Atrogin-1 is a muscle specific E3 ubiquitin ligase that facilitates atrophic signaling in cardiomyocytes (80). It and other E3 ubiquitin ligases appear to be upregulated in cardiomyocytes by treatment with DOX and/or other anthracyclines (81, 82). This leads to the degradation of contractile proteins, and reduction in cardiomyocyte size (81). As cardiomyocyte size is related to overall contractile force generation, reduced cardiomyocyte size (atrophy) compounds reduced cardiomyocyte numbers in diseased states and eventually manifests as progressive reduction in cardiac function, as in the case of DOX-induced cardiotoxicity (83).

## Therapeutic Strategies for Preventing or Treating Anthracycline Cardiotoxicity

At present, monitoring and screening for cardiotoxicity following anthracycline therapy is imperative for timely treatment. In a recent prospective study of anthracycline-treated patients, of the 9% of patients who developed heart failure, 98% of cases occurred within a year following anthracycline treatment (40). Countless prophylactic therapeutics are currently under study in animal models but few have been assessed clinically. Furthermore, only a small number cardioprotective therapeutics that have been tested in humans reduce the cardiotoxic effects of anthracyclines and currently no clear guidelines or worldwide accepted therapies exist. β-blockers, angiotensin converting enzyme (ACE) inhibitors, statins, and dexrazoxane (DRZ) are drugs that have been assessed in small trials for protection against anthracycline cardiotoxicity. Results are promising, but data is limited by small study sizes and variability in study methods such as follow up time. Early identification of reduced cardiac function and immediate treatment with heart failure medication such as enalapril (an ACE inhibitor) alone or in combination with β-blockers (carvedilol, or bisoprolol) provided either full or partial improvement of cardiac function in 82% of patients (40). A small group of non-Hodgkins lymphoma patients receiving ramipril and/or bisoprolol as a prophylactic during anthracycline treatment also exhibited reductions in new symptoms of cardiotoxicity and projected prolonged survival (84).

Statins are commonly prescribed to reduce morbidity and mortality associated with atherosclerosis. Given the cardioprotective nature of statins, and the fact that cancer patients receiving chemotherapy may also be concurrently treated with statins for atherosclerosis, the prophylactic effect of statins on anthracycline cardiotoxicity has been examined in a number of small trials. Breast cancer patients on continuous statin therapy had reduced risk of heart failure following anthracycline treatment compared to those with non-continuous statin therapy (85), and similarly, anthracycline induced decline in left ventricular ejection fraction (LVEF) was reduced by statin treatment as compared to no statin treatment (86). In a small study of 40 patients, those receiving atorvastatin prior to chemotherapy infusion showed no significant change in LVEF at 6 months post therapy compared to a reduction in LVEF in those receiving anthracycline alone (87).

While statins and ACE inhibitors have shown promising results in mitigating resultant anthracycline cardiotoxicity or preventing cardiotoxicity when individuals happen to be taking them concurrently with chemotherapy for treatment of comorbidities, DRZ is currently the only United States Food and Drug Administration, and Health Canada approved prophylactic drug for use in combination with DOX to specifically limit cardiotoxicity in adults (88, 89). DRZ acts as an iron chelator, interferes with ROS production, can bind to Top-2β to inhibit complex formation with DOX, and also reduce Top-2β expression (90–92). Early multi-center randomized double blind trials of breast cancer patients receiving combination chemotherapy which included a cumulative DOX dose of 300 mg/m^2^ reported a hazard ratio of cardiac events of 2.63 (placebo vs. DRZ); however, despite this promising effect of DRZ on heart function, patient survival was not improved (93). A systematic review also identified an association of DRZ with reduced risk of cardiovascular complications, but increased risk of secondary malignant neoplasms in children receiving chemotherapy (94). Given these results, Health Canada cautions against use of DRZ in children, as well as in elderly populations with reduced cardiac, hepatic, or renal function.

Identification of a treatment that protects against the cardiotoxic side effects without impacting the chemotherapeutic effects of DOX remains of utmost importance. Potential for development or further study of effective primary prevention therapies exists given the expansion of research uncovering the broad nature of mechanisms in the pathogenesis of anthracycline mediated heart failure.

## HDL and Role in Cancer and Cancer Therapy

HDL has long been associated epidemiologically with reduced risk for cardiovascular disease. The main mechanistic explanation has traditionally been its apparent protection against atherosclerotic narrowing of arteries, thereby combating ischemic cardiovascular disease. However, recent research in pre-clinical models have increasingly suggested that HDL may exert direct cardioprotective effects on the heart itself (24). Furthermore, HDL based nanospheres have been developed as delivery vehicles for a variety of drugs including chemotherapeutic agents. Finally, pre-clinical studies have shown that delivery of supra-physiological amounts of HDL's major apolipoprotein, ApoA1, may itself attenuate tumor growth. In the following sections, we provide an overview of HDL structure, composition, formation and function and then discuss recent findings demonstrating direct cardioprotective effects of HDL and/or its precursor, ApoA1 against DOX-induced cardiotoxicity, advances in the use of HDL based nanospheres for encapsulation of DOX for therapeutic delivery and direct anti-tumor effects of ApoA1.

### HDL Structure and Composition

Lipoproteins are diverse biological particles that provide a means of transport for lipids between cells, tissues, and other lipoproteins, and can activate intracellular signaling pathways (95). Lipoproteins are separated into five classes (chylomicron, VLDL, IDL, LDL, and HDL) based on criteria including density, size, and relative content of lipids (cholesterol and triglyceride), and apolipoproteins (95). HDL represents one of the five major classes of lipoproteins, distinguishable from others based on their small particle size (5–11 nm), high density (1.063–1.21 g/ml), and unique apolipoprotein content (96). Unlike other lipoproteins, HDLs are unique in their cytoprotective actions and initiate anti-oxidative, anti-apoptotic, and anti-inflammatory effects. HDL can inhibit the oxidation of LDL and enhance endothelial function by inhibiting the expression of endothelial adhesion molecules (97). In addition, HDL can suppress atherosclerosis progression and inflammation by modulating production of monocytes and neutrophils (98).

HDL represents a class of particles of distinct protein and lipid composition. The proteome of HDL is diverse and can contain close to 100 proteins, more than can fit on a single particle, underscoring the notion that HDL represents a class of particles of distinct compositions. The HDL proteome includes apolipoproteins, diverse enzymes, lipid transfer proteins, acute phase proteins, and proteinase inhibitors, and other proteins of distinct functions (99). ApoA1 (243 amino acids, 28 kD) is the most abundant protein in HDL, comprising 70% of the protein carried by HDL (100). ApoA1 has over 90% amphipathic α-helical content, allowing for formation and stabilization of the HDL (101). ApoA1 is linked to several beneficial effects of HDL. Therefore, several research groups have reported that HDL quality is highly dependent on the abundance and function of ApoA1 (102).

### HDL Formation and Function

ApoA1 is secreted by the liver (70%) and small intestine (30%) in a lipid-poor state, and is assembled into HDL by the addition of phospholipids and unesterified cholesterol aided by the ATP-binding cassette transporter A1 (ABCA1) at the cell surface, forming nascent HDL (an immature form of HDL). Next, the nascent HDL particle becomes mature HDL by activating lecithin: cholesterol acyltransferase (LCAT), which converts unesterified cholesterol to cholesteryl esters. The esterification of cholesterol increases its hydrophobicity, resulting in its movement into the core of the HDL particle and the particle itself adopting a spherical shape (103, 104).

HDL particles are continuously remodeled and catabolized by plasma and membrane proteins, thereby giving rise to dynamic subfractions. Membrane receptors including ATP-binding cassette transporter G1 (ABCG1) and the scavenger receptor class B type 1 (SR-B1) promote movement of lipids between cells and HDL, and plasma proteins such as cholesteryl ester transfer protein (CETP) assist in movement of lipids between lipoproteins. SR-B1 is a high affinity receptor for HDL, is highly expressed in liver and steroidogenic tissues, and plays a critical role in tissue uptake of HDL cholesterol, a key step in reverse cholesterol transport, which is the transport of cholesterol from peripheral tissues to the liver for repackaging into nascent lipoproteins or excretion (105). ApoA1 appears to play an important role in this process as adenoviral mediated or transgenic overexpression of human ApoA1 in mice leads to enhanced reverse cholesterol transport (106).

### HDL Targeted Therapeutics

The Framingham Heart Study shows strong relationships between levels of HDL and the incidence of developing heart disease (25). Statins, inhibitors of hydroxymethylglutaryl coenzyme A (HMG-CoA), a key enzyme in the cholesterol biosynthetic pathway, have long been major therapeutic tools in the reduction of cardiovascular events. This is due to their ability to reduce production and increase clearance of VLDL and LDL, thereby reducing blood cholesterol levels. Many statins have also been shown to modestly raise HDL cholesterol levels by between 3 and 15% (107–109). The mechanisms and clinical benefit of statin triggered increases in HDL cholesterol are unclear and still the subject of debate (107–111). In part this may be due to the difficulty in evaluating the contribution of the relatively modest statin-induced increases in HDL-cholesterol levels in the context of dramatic reductions in LDL cholesterol.

The epidemiological association of increased HDL cholesterol with reduced risk for cardiovascular disease has, over the years, led to efforts to increase HDL levels pharmacologically. The focus, however, has been in increasing HDL-cholesterol as opposed to increasing HDL particles or, more subtly, to increasing functional HDL particles. This focus on raising HDL cholesterol levels has led to the development of CETP inhibitors which block the CETP mediated transfer of cholesterol from HDL to triglyceride rich lipoproteins such as VLDL. These drugs increase HDL cholesterol levels by more than 25% (up to 60%), but largely have not reduced cardiovascular events (107, 109, 112–118). More recent attempts at increasing HDL particle number and/or function have focused on infusion of reconstituted HDL, administration of ApoA1 mimetics, or upregulation of ApoA1 production by liver (119–124). CSL112 is a new reconstituted HDL (rHDL) made with human ApoA1. Clinical trials of CSL112 showed that it enhances cholesterol efflux capacity an important measure of HDL mediated reverse cholesterol transport mediated cardiovascular protection (124, 125). Also, the AEGIS-I trial suggests that CSL112 has advantages over other rHDL formulations (e.g., CSL-111, CER-001) or ApoA-I Milano because it is well-tolerated with no side effects in major organs (such as liver or kidney toxicity) or immunogenicity (126–128). However, the potential benefit of CSL112 in reducing major adverse cardiovascular events in this group of high-risk patients still remains to be shown in the large phase III AEGIS-II study that is expected to be concluded in 2022 (124, 125).

### Preclinical Studies of HDL Effects on DOX-Induced Cardiotoxicity

**Figure 3:**
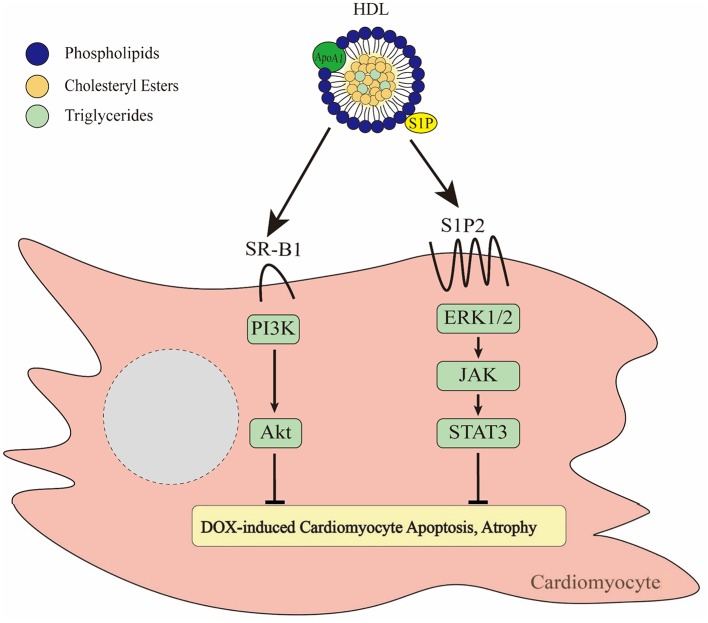
Effects of HDL on DOX-induced cytotoxicity of cardiomyocytes. HDL binds to SR-B1 leading to activation of the PI3K/Akt pathway (137). In addition, HDL is able to activate ERK1/2-JAK-STAT3 signaling via a pathway involving HDL mediated delivery of the bioactive lipid, S1P acting via the S1P2 receptor (136). Together these pathways prevent DOX-induced apoptosis and atrophy.

The protective effects of HDL against cardiovascular disease have long been the subject of intensive research. While most of the focus has been on the ability of HDL to protect against atherosclerosis and vascular dysfunction, and the impacts of that on ischemic cardiovascular disease, more recent focus has increasingly been placed on the direct cardioprotective effects of HDL, through its interactions with cardiomyocytes themselves (24). Epidemiological studies, as well as pre-clinical studies in animal models, have demonstrated that HDL can protect against cardiac disease independent of effects on coronary artery atherosclerosis, suggesting that it may also exert direct effects on the heart itself (129–132). For example, HDL treatment has been shown to protect hearts (*in vivo* and *ex vivo*) and isolated cardiomyocytes, from ischemia/reperfusion injury and infarction (130–132). In the context of DOX-induced cardiotoxicity, recent studies using isolated cardiomyocytes in culture (133–136) and in preclinical animal models (135, 137) demonstrate that HDL is able to protect against cardiomyocyte apoptosis and myocardial atrophy. For example pre-treatment of cultured cardiomyocytes with HDL prior to subsequent treatment with DOX, protects them against DOX induced apoptosis (137, 138). Similarly, HDL pretreatment of cultured cardiomyocytes protects them against other stresses leading to cytotoxicity, including necrosis resulting from oxygen and glucose deprivation (134), suggesting that HDL may protect cardiomyocytes against diverse forms of cell death. HDL mediated protection against DOX-induced cardiomyocyte apoptosis has been reported in different studies to involve the activation of AKT (134, 137) or the activation of the signal transducer and activator of transcription (STAT) 3 (138). Pharmacological or genetic inhibition of these signaling mediators has been shown to impair HDL mediated protection of cardiomyocytes against DOX-induced apoptosis (137, 138). The importance of the AKT pathway in cardioprotection against DOX has also been demonstrated by the finding that expression of constitutively active AKT1 in the myocardium inhibits DOX induced cardiotoxicity by preventing left ventricular dysfunction and cardiac atrophy (139). Furthermore, cardiac restricted overexpression of STAT3 in mice led to protection against DOX-induced atrophy and congestive heart failure, whereas cardiac specific knockout of STAT3 in mice was accompanied by increased cardiac fibrosis and age-dependent heart failure (140, 141). AKT and STAT3 form the respective cornerstones of the RISK (reperfusion injury salvage kinase) and SAFE (survivor activating factor enhancement) signaling pathways known to play important roles in cardioprotection, for example in the setting of ischemia/reperfusion injury (142, 143). These data also suggest that AKT and STAT3 are critical mediators of cardioprotection against DOX-induced cardiotoxicity (Figure 3). HDL dependent activation of AKT and STAT3 and other signaling pathways has been reported in different cell types to involve HDL mediated delivery of the bioactive lipid, sphingosine-1-phosphate (S1P), acting via the S1P receptors (136, 144–148) (Figure 3). In the case of cardiomyocytes, HDL and S1P mediated activation of STAT3 appears to be mediated by the S1P receptor 2 (S1PR2) (136). On the other hand, the involvement of S1P/S1P receptors in HDL mediated activation of AKT signaling in cardiomyocytes has not been demonstrated (134, 135, 137). However, HDL mediated activation of AKT signaling in cardiomyocytes and protection of cardiomyocytes against DOX-induced apoptosis appears to require the HDL receptor, SR-B1. SR-B1 is expressed by both mouse and human cardiomyocytes in culture and mouse cardiac tissue (137, 149). The ability of HDL to induce AKT phosphorylation and protection against DOX induced cytotoxicity in cultured mouse or human cardiomyocytes was lost when the gene for SR-B1 was either knocked out or knocked down (135, 137). SR-B1 mediates lipid transport between bound HDL particles and cells via a hydrophobic channel, suggesting that SR-B1 mediated transport of HDL bound, water insoluble S1P molecules from HDL, into the cell membrane, where they can access S1P receptors may be a potential mechanism for the involvement of SR-B1, and HDL associated S1P and S1P receptors in cardioprotection. This, however, remains to be demonstrated experimentally. The potential role for HDL associated S1P in cardioprotection against DOX-induced cardiotoxicity highlights the importance of understanding the role of HDL composition in evaluating HDL function and designing HDL based therapeutics such as reconstituted HDL-like particles (150).

These *in vitro* studies of HDL mediated protection against DOX-induced cardiotoxicity have recently been extended to *in vivo* models (135, 137) by examining the effects of increased circulating HDL levels on DOX-induced cardiotoxicity in mice. We first tested the effects of genetic overexpression of human ApoA1, on cardiotoxicity induced by repeated weekly DOX dosing in mice. Overexpression of transgenic human ApoA1 in mice has been shown to trigger dramatically increased circulating HDL levels by seeding the formation of new mature HDL particles (151). In one study, transgenic overexpression of human ApoA1 in mice virtually completely prevented chronic low dose DOX treatment from triggering myocardial apoptosis and atrophy, and protected mice from DOX-treatment induced reduction in left ventricular function (137). A drawback of this study was that although it represented a proof of concept, transgenic overexpression of ApoA1 led to levels of ApoA1 and HDL that were extremely high and therefore not likely to be therapeutically relevant (137). A more recent study, however, demonstrated that intraperitoneal injection of purified ApoA1 similarly prevented cardiotoxicity associated with chronic low dose DOX treatment in mice (135). Mice that were treated with five weekly injections of DOX alone exhibited substantial apoptosis in cardiomyocytes in hearts, and substantially reduced left ventricular function, whereas control mice that did not receive DOX displayed little myocardial apoptosis and normal left ventricular function (135). On the other hand mice that were treated with injection of ApoA1 alongside DOX were virtually completely protected against DOX-induced myocardial apoptosis and left ventricular dysfunction (135). Regardless of means of HDL increase (ApoA1 transgenic expression or ApoA1 injection) cardioprotection was lost if mice lacked SR-B1 (135, 137). In fact, SR-B1 knockout mice were more susceptible to DOX induced cardiotoxicity than corresponding wild type mice. This effect of SR-B1 appeared to be associated with SR-B1 expression in cardiac tissue, consistent with observations that SR-B1 expression in cultured cardiomyocytes was required for HDL mediated protection against DOX-induced apoptosis (135, 137). These findings clearly demonstrate that in pre-clinical models, HDL-therapies such as injection of the HDL precursor ApoA1 have the potential to protect against DOX induced cardiotoxicity but are dependent on the expression of cardiomyocyte SR-B1 (Figure 3).

### HDL Based Delivery of Chemotherapeutics

In addition to HDL's ability to protect cardiomyocytes against cytotoxicity induced by anti-cancer agents, reconstituted HDL (rHDL)-based nanoparticles have also been explored as drug delivery vehicles for chemotherapeutic agents such as DOX. The use of rHDL as a drug delivery system for DOX has been studied using both *in vitro* and *in vivo* methods. Yuan et al. showed that DOX encapsulated in HDL particles (rHDL-DOX) is more efficiently taken up by and more effective at inducing apoptosis in hepatocellular carcinoma cells, when compared to DOX alone or encapsulated in liposomes (45). Furthermore, in preclinical mouse tumor models, treatment with rHDL-DOX resulted in greater tumor regression than DOX alone (45). Wang et al. confirmed that incorporation of DOX into rHDL-based particles enhanced the cytotoxic effects of DOX on tumors *in vivo* and cancer cells *in vitro* (152). Furthermore, they demonstrated that the HDL receptor SR-B1 was required in tumor cells for rHDL mediated delivery of the encapsulated DOX (152). Interestingly, the authors measured DOX tissue distribution after treating mice with rHDL-DOX and showed that DOX uptake by the heart was low (152). Others have tested the effects of using rHDL to deliver paclitaxel (PTX) either alone or in combination with DOX. Co-delivery of PTX and DOX encapsulated in rHDL was shown to improve their anti-cancer effects over co-administration of non-encapsulated PTX and DOX (153). When used to treat preclinical models of liver cancer, the majority of PTX and DOX delivered via rHDL was found in the liver tumors (attributed to uptake via SR-B1) with little accumulation in the heart and very little cardiac damage (153). These findings suggest that, at least for liver cancer rHDL encapsulation can provide a means for targeted delivery of anti-cancer agents to tumor cells, sparing cardiac tissues. Whether the reduced cardiac damage was solely due to targeted delivery of the anti-cancer agents to the hepatic tumor over the heart or whether it also involved induction of survival signaling at the heart (PI3K/AKT and STAT3 signaling as described above) remains to be determined. It also remains to be determined whether rHDL-mediated chemotherapeutic delivery is effective against other types of cancer or against tumor cells which do not express high levels of SR-B1. Nevertheless, these studies suggest the potential for rHDL based drug delivery systems to confer tissue selective delivery to at least some types of tumors, sparing the heart from cardiotoxic damage. More research is required to determine the full potential of this.

### HDL and Cancer

In addition to research showing that HDL can protect cardiomyocytes from chemotherapy-induced cytotoxicity both directly by inducing survival signaling in the cardiomyocytes, and indirectly by acting as a targeted delivery system for anti-cancer agents, sparing the heart, other research has suggested that HDL and its precursor ApoA1 may also have direct anti-tumor effects themselves.

#### Endogenous HDL and Cancer Risk

Results of epidemiological studies of endogenous HDL cholesterol levels and the incidence of cancer are mixed with some studies reporting an inverse correlation between HDL cholesterol and cancer risk and/or mortality, while other studies report minimal association, particularly when corrected for confounding factors (152, 154–159). Contributing to this is uncertainty over the cause-vs.-effect relationship between low HDL-cholesterol and cancer, with some studies suggesting that tumor cells may drive the lowering of HDL-cholesterol levels by utilizing HDL-cholesterol to support tumor growth (159). Complicating matters further are reports that HDL prepared from cancer patients or from patients with other co-morbidities, such as type 2 diabetes or obesity exhibit altered functions as compared to HDL from unaffected individuals, for example, promoting rather than inhibiting migration and invasion of tumor cells in *in vitro* assays (160–166). Therefore, it is presently unclear what, if any, effects levels of endogenous HDL or variations in those levels have on cancer development.

#### Anti-cancer Therapeutic Potential of the HDL Precursor ApoA1

On the other hand, preclinical studies in mouse models have suggested that supra-physiological levels of ApoA1 may have therapeutic potential against tumor growth and metastasis. For example, Zamanian-Daryoush et al. reported that transgenic overexpression of human ApoA1 reduced, while complete knockout of endogenous ApoA1 increased tumor growth and metastasis in mice compared to control mice with normal levels of endogenous ApoA1 (167). They also demonstrated that pharmacological treatment with purified ApoA1 similarly attenuated both primary tumor development and metastasis in mouse models (167). They provided evidence that ApoA1 reduced tumor angiogenesis and recruited tumor cell targeting macrophages and CD8^+^ cytotoxic T cells, thereby altering the tumor microenvironment to one less permissive for tumor development (167, 168). By using different tumor cell lines, including a human melanoma cell line (A375), they demonstrated that supra-physiological levels of ApoA1 may have general anti-neoplastic effects including toward human tumors (167, 168). Others have reported that synthetic ApoA1 mimetic peptides, which replicate the amphipathic properties of ApoA1, also exhibit anti-tumor properties, when used at pharmacological concentrations. For example, the ApoA1 mimetic peptide, L-5F was reported to prevent angiogenesis, suggesting that it may have therapeutic potential against angiogenesis associated diseases such as cancer (169). In preclinical studies, the ApoA1 mimetic peptide 4F has been reported to suppress ovarian tumorigenesis (170). Similarly, preclinical studies demonstrated that the recombinant ApoA1 mimetic peptide 6F reduced tumor burden in mouse models of metastatic lung cancer (171). However, ApoA1 mimetic peptides may exert anti-tumor effects via mechanisms distinct from ApoA1. For example ApoA1 mimetic peptides are thought to strongly bind and neutralize lysophosphatidic acid (LPA), which is known to stimulate cell proliferation, oncogenesis, and metastasis (172). On the other hand, neither transgenic overexpression of human ApoA1 nor ApoA1 knockout affected LPA levels in tumor-bearing mice (167). Whether ApoA1 (injected or overexpressed) or ApoA1 mimetic peptides exert anti-tumor effects by driving the increased formation of HDL-like particles or whether their anti-neoplastic effects are independent of HDL particle formation has not been examined. Other pre-clinical studies have reported that in certain cases, HDL may drive the development of breast cancer, particularly in circumstances when breast tumor cells overexpress the HDL receptor, SR-B1, since this receptor can mediate both survival signaling and uptake of cholesterol fueling tumor growth (173–177). Therefore, direct anti-tumor effects of ApoA1 or ApoA1-mimetic peptides may be restricted to tumors that do not overexpress SR-B1; although SR-B1 overexpression in tumors could be exploited by strategies that encapsulate chemotherapeutic agents like DOX in HDL based nanoparticles (see previous section), which may be readily and preferentially taken up by tumor cells overexpressing SR-B1.

## Conclusions

**Figure 4:**
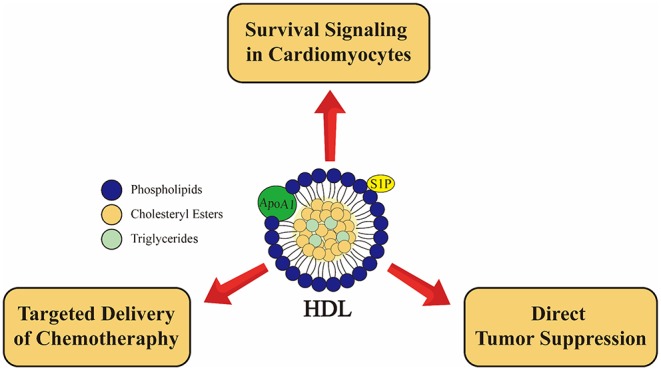
Emerging roles of high density lipoprotein (HDL) in cancer and cancer therapy associated cardiotoxicity from preclinical studies. HDL induces cardioprotective effects in cardiomyocytes via SR-B1 receptor and activation of PI3K-Akt pathway as well as via S1P2 receptor leading activation of STAT3-JAK-ERK1/2 pathway (136, 137). rHDL used as a drug delivery system may allow for targeted delivery to at least some types of tumors, sparing cardiomyocytes (45, 152). Furthermore, the major apoliprotein of HDL (ApoA1) directly attenuates tumor growth and metastasis in preclinical models (167). These pathways are not necessarily muatually exclusive.

Preclinical studies suggest that HDL targeted therapies involving pharmacological treatment with supra-physiological levels of ApoA1, peptides based on ApoA1 (ApoA1 mimetic peptides) or rHDL like particles may show promise in the protection against chemotherapy related cardiotoxicity via a number of mechanisms (Figure 4). These include (1) direct HDL mediated survival signaling in cardiomyocytes leading to protection against cytotoxicity, as exemplified by studies using DOX as a cardiotoxic agent; (2) indirect protection afforded to the heart by utilizing rHDL-based nanoparticles as targeted delivery vehicles for chemotherapeutic agents which spare the heart and have the potential to target tumor cells which may overexpress SR-B1; and (3) indirect protection resulting from direct ApoA1 mediated tumor suppression (Figure 4). It remains to be demonstrated experimentally whether these mechanisms broadly impact diverse malignancies and chemotherapeutic agents or are specific for those that have been tested to date. Importantly, these mechanisms may not necessarily be mutually exclusive. For example, in the case of treatment with ApoA1 along with chemotherapeutic agents such as DOX, the ApoA1 may be acting by seeding the formation of new HDL particles which may incorporate the chemotherapeutic agent, act as targeted delivery systems for certain types of tumors, directly attenuate tumor growth, and directly induce survival signaling in cardiomyocytes, thus inducing both direct and indirect mechanisms of cardioprotection simultaneously. However, studies need to be designed to test whether these mechanisms do occur simultaneously, in the same preclinical models. Whether or not they do occur simultaneously, much more work remains to be done do determine the full potential for HDL targeted therapies as therapeutic approaches to prevent chemotherapy induced cardiotoxicity in human disease.

## Author Contributions

All authors listed above made substantial, direct and intellectual contributions to the work, and have approved the final version.

## Conflict of Interest

The authors declare that the research was conducted in the absence of any commercial or financial relationships that could be construed as a potential conflict of interest.
